# Demographic Characteristics, Motivation and Perception of Change as Determinants of Memory Compensation Self-Reports After Acquired Brain Injury

**DOI:** 10.3389/fpsyg.2021.607035

**Published:** 2021-07-14

**Authors:** Sophie Martin, Draushika Mooruth, Estelle Guerdoux-Ninot, Clémence Mazzocco, Denis Brouillet, Laurence Taconnat, Raphaël Trouillet

**Affiliations:** ^1^Laboratoire EPSYLON EA 4556, Paul Valéry University Montpellier 3, Montpellier, France; ^2^Cogithon, Participative Innovations Platform Promoting Human Knowledge and Solutions When Facing Disabilities, Maison des Sciences de l’Homme “Les Sciences Unies pour un autre Développement,” FR 2005 du CNRS, COMUE Languedoc-Roussillon Universités, Montpellier, France; ^3^Department of Supportive Care, Unit of Psycho-Oncology, Montpellier Cancer Institute (ICM), Montpellier, France; ^4^UMR 1302 Institute Desbrest of Epidemiology and Public Health, INSERM, Univ Montpellier, Montpellier, France; ^5^Université de Tours, Université de Poitiers, UMR 7295 Centre de Recherches sur la Cognition et l’Apprentissage, Poitiers, France

**Keywords:** memory compensation strategies, self-report, brain injury, motivation, change, rehabilitation

## Abstract

**Introduction:**

Individuals with brain injuries experience cognitive and emotional changes that have long-lasting impacts on everyday life. In the context of rehabilitation, surveys have stressed the importance of compensating for memory disturbances to ease the impact of disorders on day-to-day autonomy. Despite extensive research on the nature of neurocognitive impairments following brain injury, few studies have looked at patients’ perceptions of these day-to-day compensations. This study examines these perceptions; in particular, what brain-injured people believe they do to compensate for memory deficiencies in everyday life. It also investigates the determinants of reported compensation strategies (age, gender, perceived stress, change awareness and motivation to succeed).

**Methods:**

Eighty patients and 80 controls completed the French Memory Compensation Questionnaire, a self-report measure of everyday memory compensation. Five forms of compensation were investigated: External and Internal strategies, Reliance on social help, and investments in Time and Effort, along with two general factors: the degree of importance attached to Success (motivation) and perceptions of Change. Participants also completed measures of demographic and emotional aspects that may affect everyday compensation perceptions.

**Results:**

The brain-injured group reported significantly more frequent use of memory compensation strategies than controls, with the exception of External aids. Large effects were observed for Reliance and Effort. Demographic, motivation and perception of change determinants were found to have different effects depending on the compensation strategy, and mediated the direct effect of brain injury on reported compensation.

**Conclusion:**

Clinical and rehabilitation neuropsychologists often seek to have a better sense of how their patients perceive their compensatory behaviors. In practice, such an understanding is needed to help select appropriate methods and improve the long-term impact of rehabilitation programs: memory rehabilitation will fail if neuropsychologists do not deal, first and foremost, with the emotional and metacognitive issues surrounding traumatic brain injury (TBI), rather than focusing on cognitive efficiency.

## Introduction

Traumatic brain injury (TBI) and stroke are known to be the most significant health problems worldwide (Tagliaferri et al., 2006). In recent decades, we have witnessed a plethora of improvements in trauma care, together with road safety and security measures that have lowered the morbidity and mortality rate following TBI or stroke. However, a large number of victims continue to experience long-lasting neuro-behavioral sequelae. Among the most commonly reported symptoms are changes in retrospective and prospective memory functioning (Goldstein and Levin, 1996; Kinsella et al., 1996; Groot et al., 2002; Louda et al., 2007; Roche et al., 2007; Fish et al., 2010; Martin et al., 2013). These difficulties clearly degrade the quality of life of victims and increase the risk of developing further disabilities in the long term (Bach-y-Rita and Bach-y-Rita, 1990; Cicerone et al., 2000).

Encouraging resilient behavior through compensatory strategies, as reported by Freund and Baltes (1998), has proved to be successful in overcoming degraded memory skills. However, intervention programs are highly specialized and costly (Bach-y-Rita and Bach-y-Rita, 1990; Cicerone et al., 2000; Brewer-Mixon and Cullum, 2013). According to the latter authors, compensation is better than retraining approaches, and is the most effective rehabilitation strategy when dealing with memory deficits. The term *compensation* refers to a set of adaptive, strategic mechanisms to promote health that are developed to overcome the loss (Bäckman and Dixon, 1992; Dixon et al., 2008). In that context, Dixon et al. (2001) have described five type of compensation strategies to support memory loss: the use of (a) external aids, (b) internal mnemonic techniques, (c) increased effort (d) extra time and (e) others as memory aids (reliance). Several studies have investigated the extent to which demographic characteristics or personality dispositions predict self-reported use of those strategies during aging. Dixon et al. (2001) explored the impact of age and gender differences in the experience of memory compensation (see also van der Elst et al., 2011; Martin et al., 2015). Older men reported greater use of external and reliance strategies than younger ones, which was not the case for women. Moreover, women reported higher motivation values than men. To go further, de Frias et al. (2003) investigated the extent to which memory compensation self-reports were related to concurrent variables such as age, gender or personality dispositions. Once again, gender proves to be a significant predictor of compensation self-reports: women reported more frequent use of external and internal strategies than men, and greater effort in remembering, whereas men relied more frequently on other people as memory aids. Furthermore, aging was associated with a greater commitment to better performance in a memory task, and increased reported use of memory compensation aids over the past 5–10 years (see also Prigatano, 1999). de Frias et al. (2003) also explored whether feeling preoccupied, stressed and anxious was linked to subjective self-ratings of memory compensation. This point is of particular interest for our concerns. There is an increased risk of developing perceived stress post-TBI. Indeed, stress is a common experience of TBI (Walsh et al., 2020). de Frias et al. (2003) demonstrated that anxiety was robustly related to an increase in self-reported use of compensatory strategies. Martin et al. (2015) confirmed that perceive stress was linked to all compensation scales except the External one during aging. Garrett et al. (2010) indicated that high-stress older compensated whether or not they perceived memory errors. Altogether, those results suggest that demographic and personality backgrounds influence reported compensation behaviors strategies designed to improve day-to-day functioning. With respect to aging, it is now clear that those variables have direct effects on what people think about their own compensatory behaviors (de Frias et al., 2003; de Frias and Dixon, 2005; van der Elst et al., 2011; Martin et al., 2015; Mazzocco et al., 2015). According to de Frias et al. (2003), those memory compensation correlates “may serve as a key to identifying important resources that may prolong functional competence and successful cognitive aging” (p. 14).

There is a large body of work dealing with the effects of TBI on cognitive functioning (Schretlen and Shapiro, 2003) or on the effectiveness of multidisciplinary rehabilitation process (Cicerone et al., 2005; Rohling et al., 2009; Tsaousides and Gordon, 2009). Some studies examined the impact of age (e.g., Goldstein and Levin, 2001; Draper and Ponsford, 2008; Senathi-Raja et al., 2010) or gender (Farace and Alves, 2000; Ma et al., 2019) on acquired lesion outcomes. Those studies highlight that outcome is worse in woman than in men for most of cognitive domains; and that older injured individuals performed worse than did younger injured individuals. But to date, there is a paucity of research that focused on the specific consequences of brain injury on people’s subjective beliefs about how they compensate. Changes in perceptions following head trauma are often studied in the light of impaired self-awareness or the denial of the disability (Roche et al., 2002). But, to our knowledge, it is not yet known whether self-perceptions of everyday compensations are related to demographic characteristics, motivation, perception of change or stress. Therefore, the question arises of whether the effect of brain damage on perceptions of compensatory strategies remains significant after adjusting for compensation correlates.

No research has specifically focused on the nature of the relationship between TBI and perceptions of compensation, but several studies of memory training in the context of brain-injured patients, aging and memory complaints are relevant. Thöne and Walther (2001) suggested a cause and effect relationship between the severity of brain injury, the use of memory compensation strategies and the ability to master everyday life in a sample of 53 brain-injured persons of different etiology. Participants that were classified as “independently living” used significantly more External supports (e.g., notebooks) and Internal memory aids (e.g., imagery) than less-autonomous patients. All data were collected through semi-standardized interviews. The authors concluded that the ability to successfully compensate for memory deficits was a relevant predictor of everyday independent functioning following brain injury. In the same vein, Prigatano and Kime (2003) investigated the use of memory compensation strategies through the Memory Compensation Questionnaire (de Frias and Dixon, 2005) in 29 patients with memory complaints following heterogeneously-acquired brain disorders (i.e., TBI, ruptured aneurysms, arteriovenous and cavernous malformations, cerebrovascular accidents, tumors, hydrocephalus, cerebral anoxia). Their study was specifically based on patients’ self-reports following memory compensation training. They found that, post-training, compared to Dixon et al.’s (2001) normative data, all patients self-reported greater use of memory compensation strategies and prolonged effort in doing so. Within the patient group, the authors did not observe any effect of employment or therapeutic alliance on compensation perceptions. However, a major limitation of Prigatano and Kime’s (2003) study is a lack of adequate experimental controls. First, there was no formal control group. The study compared compensatory perceptions in a patient group aged 26–60 (16 males and 13 females), with normative data from Dixon et al.’s (2001) sample that only included males aged 58–64. As mentioned above, gender and age change one’s perception of one’s own compensatory behavior. Secondly, comparisons were descriptive and no further inferential analyses were conducted. Therefore, there is a lack of support for the hypothesis that brain-injured patients differ from a control group with respect to their perceptions of compensatory behavior. With the current data, it is difficult to conclude whether having sustained an injury changes these self-perceptions. Third, the results are limited because compensatory strategies used by the sample were expressed, and hence compared, only after training. Therefore, it is unclear whether perceptions differed between the group of brain-injured patients and the matched control population before training. One objective of our research was to address this issue.

In sum, numerous studies have focused on the impact of brain damage on memory efficiency, but few have investigated compensatory approaches, and even fewer have implemented a standardized assessment of victims’ perceptions of their own day-to-day behaviors (Prigatano and Kime, 2003; Huckans et al., 2010; Shum et al., 2011). It is clear that asking a patient’s opinion about his or her own compensatory behavior may not reflect their actual functioning, while factors such as age, gender, perceived stress, motivation and perception of change may also play a role. Therefore, the present exploratory study investigated interrelationships between self-reported compensation, brain lesion, demographic factors, motivation and perception of change. Given the findings of earlier work into aging and memory complaints following brain injury, we hypothesized that the link between self-reported compensation and brain injury would be mediated by motivation to succeed, perceptions of change and perceived stress. Moreover, we predicted that age and gender would be additional covariates in compensation patterns.

## Materials and Methods

### Participants

Eighty volunteers with acquired brain injury (67 males and 13 females) were recruited through injury associations and centers specialized in acquired brain injury rehabilitation. These associations are in charge of the social support of patients toward socio-professional reintegration. The professional workers are not allowed to directly access to the patients’ history and medical files. Only the rehabilitation physician of the intervention teams was authorized to consult the medical file. Therefore, he was in charge to confirmed the clinical diagnosis and the eligibility of the patients for our study and to check inclusion/exclusion criteria. Inclusion criteria were (1) a diagnosis of mild brain damage evaluated by an initial moderate Glasgow Coma Scale score (GCS between 9 and 12) and documented in the medical record following the period of hospitalization (according to the Diagnostic and Statistical Manual of Mental Disorders, 4th Edition, diagnosis of Cognitive disorders), (2) living independently, (3) having sustained the injury more than 6 months prior to inclusion and, finally, and (4) having residual memory problems. Exclusion criteria were drug or alcohol consumption, psychiatric disorders or having participated in a dedicated memory rehabilitation program.

Altogether, 80 patients, aged from 19 to 55 years (*M* = 35.6; *SD* = 9.8), were assigned to the Brain Injury Group (BIG). Time elapsed since the onset of brain injury ranged from 18 to 72 months, thus all victims were post-acute (see Table 1 for descriptive statistics).

**TABLE 1:** Descriptive characteristics of the Brain Injury Group (from 5 for primary education to 1 doctoral degree).

	Brain Injury Group	
Age	*N*	*N* (%)
<20	1	1.25
20–29	26	32.5
30–39	17	21.25
40–49	29	36.25
50–55	7	8.75

**Level of education**	***N***	***N* (%)**

5	44	55
4	29	36.25
3	6	7.5
2	1	1.25
1	0	0

**Marital status**	***N***	***N* (%)**

Single	39	48.75
Split/divorced	10	12.5
Divorced with children	10	12.5
Couple	7	8.75
Couple with children	14	17.5

**Months since injury**	***N***	***N* (%)**

<18	0	0
18–23	12	13.75
24–29	10	12.5
30–35	6	7.5
>36	53	66.25

Patients’ responses to questions about their memory difficulties were analyzed to assess their level of awareness of their problems relative to the clinician’s interview. All self-reported residual memory problems. Patients also underwent an initial neuropsychological examination. Psychometric measures included the forward and backward Digit Span Test that assesses the ability of a participant to recollect a string of nominal digits (WAIS, 3rd Edition). The average standard score for forward recall was 7.66 (*SD* = 1.86), and 5.48 for backward recall (*SD* = 1.88). Patients also completed the Letter–Number Sequencing task, which required them to recall a series of numbers in increasing order, and letters in alphabetical order (WAIS, 3rd Edition). The average standard score was 7.02 (*SD* = 2.74). The Free and Cued Selective Reminding Test (Buschke, 1984; Grober and Buschke, 1987) was used to develop a clinical diagnosis of episodic memory impairment. Participants learned 16 items belonging to 16 semantic categories using an encoding procedure. This was followed by three, free recall trials each lasting 2 min, followed by semantic cuing of items that were not spontaneously recalled. After a 20-min break, a delayed recall trial was run with free, then cued, recall. The average raw scores for the three free recall trials and total free recall were, respectively, 7.36 (*SD* = 2.11), 9.2 (*SD* = 2.87), 10.38 (*SD* = 2.98), and 10.64 (*SD* = 3.29).

Finally, in order to estimate potential intellectual deficit, we used the Binois–Pichot Vocabulary Test (BPVT) and the D48 Dominoes Test of general intelligence (D48). In the BPVT, participants must identify which of the six proposed words is closest in meaning to the target word. The total score is out of 44, which is translated into IQ by equivalence. The D48 test investigates the person’s non-verbal ability to draw inferences from dominoes and does not depend on verbal skills or culture. These two tests are usually used together and results are highly correlated in healthy subjects. Typically, the processes mobilized in the vocabulary test are more resistant to pathological damage. A weakness in one or both tests tends to suggest reduced overall efficiency. The average standard BPVT score (IQ by equivalence) was 94.48 (*SD* = 10.61) and 109.54 (*SD* = 12.93) for the D48 test (IQ by equivalence).

The control group (CG) consisted of 80 individuals (59 males and 21 females), aged 18–60 (*M* = 35.51; *SD* = 12.6). Participants assigned to the CG and the BIG group were statistically matched for age, gender and education. The CG was recruited through advertisements in the laboratory’s newsletter, associations, at the university, local councils, personal requests and contacts with seniors’ associations. Volunteers were encouraged to talk about the study to their friends and family. In order to acknowledge their investment, a member of our laboratory held a conference on memory and aging. Participants had no history of motor or cognitive impairment, or any neurological or psychiatric disease (i.e., epilepsy, stroke, brain injury, tumor, or cancer). As no medical records were available, and data were exclusively self-reported, a cognitive impairment screening test (the clock-drawing test) was administered (Paganini-Hill et al., 2001; Paganini-Hill and Clark, 2007; van der Elst et al., 2011). All members of the CG scored normally on this test.

The two groups were matched for age [*t*(79) = 0.04, *p* = 0.97] and education [*t*(79) = 1.69, *p* = 0.10]. Although the male population was slightly bigger in the clinical condition, the difference was not significant [χ^2^ = 12.50, NS].

### Materials

The first set of questionnaires were administered to all participants in the same order, either at local meetings or at home, during a session that lasted approximately 1 h. The first page outlined the aim of the study and the procedure in detail. Participants were asked to read and sign the included consent form prior to participation. Once sociodemographic data had been recorded (date and location of testing, date of birth, gender (forced binary choice), current or last employment, educational level, and marital status), participants completed several self-administered questionnaires in the following order:

#### The French Memory Compensation Questionnaire

The Memory Compensation Questionnaire (MCQ) is a valuable and sensitive tool designed to evaluate individuals’ beliefs concerning their compensatory behaviors in naturalistic settings (Dixon et al., 2001; van der Elst et al., 2011; Melèndez et al., 2013; Martin et al., 2015; Mazzocco et al., 2015). Martin et al. (2015) proposed a standardized, validated and normalized version of the MCQ for the French population (fMCQ). A brief version (Brief-MCQ) dedicated to aging is also available (Mazzocco et al., 2015). The present study used the full French version, since to the best of our knowledge, it is the only life span questionnaire based on normative data from a sample of 749 individuals aged 18–92.2 (*M* = 43.5; *SD* = 19.77). The following description is partly drawn from Martin et al. (2015).

The MCQ’s seven scales investigate the use of five strategies and two aspects of everyday memory compensation. The original seven-factor structure (Dixon et al., 2001) was replicated for the French version using a Confirmatory Factorial Analysis (Martin et al., 2015). The first three scales relate to the substitution mechanism found in compensation theory (Dixon et al., 2008), which consists of replacing a declining capacity by a new one, or doing something in a different way. The *External* scale (F1) contains eight items regarding the use of external aids and devices to support remembering (e.g., “Do you use shopping lists when you go shopping?”); the *Internal* scale (F2) contains 10 items regarding the use of mnemonic strategies to facilitate or improve memory efficiency (e.g., “When you want to remember the name of a person do you try to associate the name with the person’s face?”); the *Reliance* (or *Recruitment*) (F3) scale contains five items regarding the recruitment of other people for memory assistance [e.g., “When you want to remember an important appointment do you ask somebody else (for example, your spouse or a friend) to remind you?”].

The next two scales relate to remediation mechanisms that require a greater investment of time and effort to adapt to, and overcome losses: the *Time* scale (F4) contains four items regarding the extent to which the respondent invests more time in performing memory tasks (e.g., “When you want to remember a newspaper article do you read it more slowly?”); and the *Effort* scale (F5) contains six items regarding the investment of greater effort when performing a memory task such as rehearsing or retrieving information (e.g., “Do you make an effort when you want to remember the time of an important meeting?”).

The last two scales investigate general aspects of memory compensation and are of major interest in this study. The *Success* scale (General Factor 1: GF1) contains five items regarding the use of strategies that reduce the mismatch between environmental demands and personal skills by adjusting goals. It assesses the extent of commitment to memory performance and the motivation to maintain a given memory competence (e.g., “When you want to remember an event that took place when you were a child, is it important for you to remember it as perfectly as possible?”). The higher the commitment, the less a person will tend to accommodate to his/her losses, as he/she will maintain the same criterion of success and sense of control. This scale evaluates motivation to succeed in a memory task and, thus, commitment and motivation to maintain and enhance memory skills (de Frias et al., 2003). Finally, the *Change* scale (GF2) contains five items regarding the extent to which the respondent is aware of changes in their efforts to compensate during the 5–10 years prior to testing (e.g., “Do you put in effort and concentrate to remember important things more or less often today compared to 5–10 years ago?”). This last scale reflects personal insight and beliefs regarding memory loss. It implies retrospective memory bias and reflects the “good all of the time” hypothesis.

Participants responded to each item (except *Change*) on a 5-point Likert scale, with the following options: 0 = never, 1 = seldom, 2 = sometimes, 3 = often, and 4 = always. The *Change* scale consisted of the following options: 0 = much less often, 1 = less often, 2 = no difference, 3 = more often, and 4 = much more often. The wording was different for one item “Do you spend more or less time learning important things today compared with 5–10 years ago (e.g., reading things more slowly or reading them more than once)?” which had the following options: 0 = much less time, 1 = less time, 2 = no difference, 3 = more time, and 4 = much more time.

Our hypotheses were that motivation, evaluated by GF1, and retrospective bias, evaluated by GF2, would mediate the relationship between TBI and perceptions of day-to-day memory compensation strategies (evaluated by F1–F5).

#### The Perceived Stress Scale

The Perceived Stress Scale (PSS) (Cohen et al., 1983) evaluates the perception of experienced stress by measuring the degree to which respondents have found their life to be uncontrollable and overloaded during the past month. The 14 items are scored on a 5-point Likert scale with the following multiple-choice options: 0 = never, 1 = almost never, 2 = sometimes, 3 = fairly often, and 4 = very often. We used the French version, translated by Quintard (1994). It takes approximately 5 min to complete.

### Statistical Analyses

**FIGURE 1:**
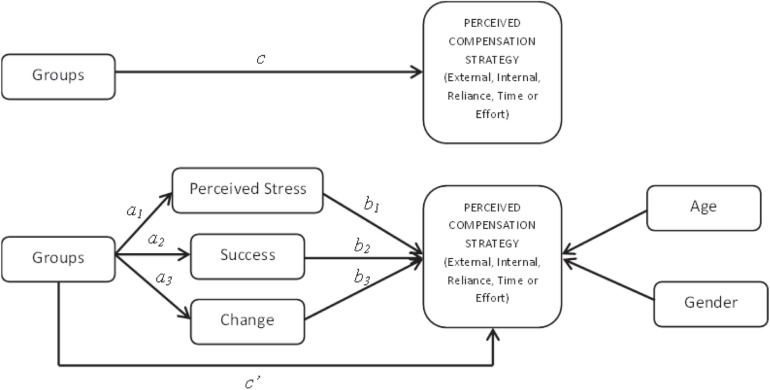
Theoretical model testing the hypothesis that brain lesion (*Groups*) has a direct effect on perceptions of compensation strategies (c), and the alternative hypothesis that it is mediated by *Perceived Stress*, *Success*, awareness of *Change* (*a_i_, b_i_, a_i_b_i_*) and demographic covariates (*Age*, *Gender*).

All data were analyzed with *R* software (R Core Team, 2013) using the *R* package lavaan (Oberski, 2014) in order to test for each of the five compensation strategies. Models that included the dummy *Group* variable were assumed to influence compensation strategies through several mediators (*Perceived Stress*, *Success*, and *Change*). Dummy variables *Age* and *Gender* were included as covariates. Each model comprised several regressions (Figure 1).

Although the number of subjects per variable is satisfactory (Austin and Steyerberg, 2015), our sample size was small (*N* = 160). Therefore, we used the Maximum Likelihood Robust estimator to calculate a robust *R*^2^ statistic and Huber-White’s robust standard errors (Maronna et al., 2006). By definition, a robust standard error is a reliable estimate of the true standard error even for non-independent and non-identically distributed (i.i.d) error terms suffering from heteroskedasticity. In addition, we used the Bentler Comparative Fit Index (*CFI*) and the Standardized Root Mean Square Residual (*SRMR*) to estimate the fit of the data, as recommended in Hu and Bentler (1999) for small sample sizes. The *CFI* statistic tests the improvement in the adjustment of the tested model to the data compared to a null model. Although a *CFI* value > 0.90 is indicative of a well-fitting model, Hu and Bentler (1999) recommend a value of 0.96 or higher. The *SRMR* is defined as the standardized difference between observed correlations and correlations predicted by the model. A value less than 0.09 is considered a good fit (Hu and Bentler, 1999). We estimated unstandardized regression coefficients (*B*), Standard Errors (*S.E.*), Critical Ratios (*C.R.*) and *p*-values. Significance was set at *p* ≤ 0.05.

As it was important to avoid any false positives (the family-wise error rate, FWER), we compared *p*-values obtained in our analyses with adjusted *p*-values (the Holm-Bonferroni stepwise method), which corrected for the problem of multiple testing and controlled for FWER. With this method, unadjusted *p*-values must be less than their adjusted values to be significant. As we ran a large number of statistical tests in our regression analyses (13 direct and indirect effects per model), we were able to calculate adjusted *p*-values. These values were inserted where *p*-values ≤ 0.05.

We recruited patients with different types of brain injury, the Brain Injury Group was therefore heterogeneous and we needed to estimate if MCQ scores would vary in function of the types of brain injury. For this purpose, in our preliminary analyses, we used the lmer function in the R package lme4 to estimate the fit of mixed-effects models (Bates et al., 2015). Mixed-effects models included both fixed and random effects. We tested random effects for the factor “pathology” (i.e., type of brain injury) because the values we observed represent a random sample from the set of all possible values. We herein estimated between-levels variance for “pathology” in the mean of the dependent variable (i.e., random intercepts) by adding random effects to the intercept. The intercept was modeled using a fixed effect parameter. For each factor of the MCQ, we subsequently competed models incorporating only the intercept as a fixed-effect with models where “pathology” was added as a random effect using Akaike Information Criterion (*AIC*) values. The model that best adjusts the data has the smallest *AIC* value.

## Results

### Inter-Group Differences for the Memory Compensation Questionnaire

Mean rating of frequency of use and standard deviation for both groups are given in Table 2. Descriptively, both groups engaged between “sometimes” and “often” in External Strategies. Persons with brain injury “sometimes” engaged in internal strategies, while this choice was less prevalent among the CG. They recruited help from others either “seldom” or “sometimes,” while this was “less than seldom” for the CG. Finally, persons with brain injury engaged *Effort* and *Time* in compensation between “sometimes” and “often,” while the CG declared somewhere between “seldom” and “sometimes.”

**TABLE 2:** Mean rating of frequency of use and standard deviation on the fMCQ.

Measure	Brain Injury Group	Control group	*t*	*p*	Adjusted *p*	Partial η^2^
**Compensatory mechanisms mean (standard deviation)**						
External *M (SD)*	2.52 (0.82)	2.33 (0.73)	1.59	0.11	0.05	0.01
Internal *M (SD)*	2.02 (0.62)	1.74 (0.55)	2.97	0.003	0.01	0.05
Reliance *M (SD)*	1.63 (0.94)	0.88 (0.64)	5.87	<0.001	0.007	0.18*
Time *M (SD)*	2.03 (0.88)	1.67 (0.80)	2.63	0.009	0.01	0.04
Effort *M* (*SD*)	2.33 (0.72)	1.70 (0.67)	5.72	<0.001	0.008	0.17*
**General factors mean (standard deviation)**						
Success *M* (*SD*)	1.90 (0.84)	1.65 (0.88)	1.79	0.07	0.02	0.02
Change *M* (*SD*)	2.74 (0.78)	2.22 (0.42)	5.19	<0.001	0.01	0.14*

* Indicates a large effect as defined by Cohen (1988) (N = 80 for both groups).

Given the above, we were interested to determine whether these self-reported patterns differed statistically between the two groups. We therefore conducted *t*-tests on each factor (Table 2). The partial eta-squared statistic compares effects by controlling for intra-subject variability with the scale taken from Cohen (1988). These tests found significant differences between the two groups of participants for all fMCQ scales, except perceived use of external aids (*t* = 1.59, NS). Specifically, persons with brain injury perceived that they used more internal strategies (*t* = 2.97, *p* < 0.01), relied more on others (*t* = 5.87, *p* < 0.001), took more time (*t* = 2.63, *p* < 0.01) and made more effort (*t* = 5.72, *p* < 0.001) than the CG.

Partial η^2^ revealed a large effect of the impact of brain injury on *Reliance* (η^2^*p* = 0.18) and *Effort* (η^2^*p* = 0.17) scales. In contrast, the impact was minimal for *Internal* (η^2^*p* = 0.05) and *Time* (η*^2^p* = 0.04) scales. Finally, *t*-tests found a significant difference between the two groups on the *Change* scale (*t* = 2.73, *p* < 0.001, η^2^*p* = 0.14), and a marginally significant difference on the *Success* scale (*t* = 1.89, *p* = 0.07, η^2^*p* = 0.02). Persons with brain injury perceived greater changes in their compensatory behaviors over the past 5–10 years, and tended to show a greater commitment to succeeding in memory tasks than the CG.

Altogether, these results highlight that self-reported compensation patterns are different for persons with brain injury compared to similar controls. To understand these differences in more detail, we explored whether the presence of a brain injury itself was sufficient to explain the effect. We predicted that the addition of characteristics related to individuals’ backgrounds would be determinant in explaining observed differences.

### Regression Equations

We estimated the effect of the types of brain injury on our results by competing models incorporating the intercept as a fixed effect with models where the random effect coding for the factor “pathology” was added. Our results revealed that our models fit incorporating only the intercept (*AIC_Extern__al_* = 588.1, *AIC_Intern__al_* = 577.50, *AIC_O__Reliance_* = 479.48, *AIC_T__ime_* = 398.10, *AIC_Effort_* = 488.44) were not improved by adding the “pathology” random effect (*AIC_Extern__al_* = 1046.15, *AIC_Intern__al_* = 1032.61, *AIC_Reliance_* = 915.21, *AIC_T__ime_* = 854.15, *AIC_Effort_* = 925.51). These results show that the random effect for the factor “pathology” is unnecessary and can be removed from our subsequent analyses.

We tested five models, one for each compensation strategy (Figure 1). Each model tested the indirect effects of *Group* on the use of each compensation strategy mediated by motivation to maintain and enhance memory skills (*Success*, GF1), personal insight and beliefs regarding memory loss (*Change*, GF2) and perceived stress (*Perceived Stress*, PSS). Each strategy was also regressed on *Age* and *Gender* as covariates. For each model, we present our results in the following order: (1) total effects (*c*_i_ paths); (2) direct effects (*a*_i_, b*_i_* and *c’_i_* paths); and indirect effects (*a_i_b_i_* values). An indirect effect is interpreted as the amount by which two cases that differ by one unit on X (IV) are expected to differ on Y (DV) through X’s effect on M, which in turn affects Y.

#### External Strategy (Model 1)

We did not observe any significant overall effect of *Group* on the perceived used of *External* strategies (*B* = 1.64; *p* = 0.08). When *Perceived Stress*, *Success* and *Change* (i.e., mediators) were added (*CFI* = 1.00, *SRMR* = 0.04), the *Group* effect was non-significant (*B* = 0.74; *p* = 0.50). We found a significant and positive effect of *Age* on the *External* factor (*B* = 0.14; *p* < 0.0001; adjusted *p* = 0.003). No significant effect was observed for *Success* (*B* = 0.17; *p* = 0.12), *Change* (*B* = 0.28; *p* = 0.12), *Perceived Stress* (*B* = −0.02; *p* = 0.83), or *Gender* (*B* = 0.99; *p* = 0.38). No significant indirect effects were found for *Change* (*B* = −0.72; *p* = 0.15), *Success* (*B* = 0.21; *p* = 0.212), or *Perceived Stress* (*B* = 0.03; *p* = 0.82).

In sum, our model indicated that only aging was consistent with a perceived increase in external strategies.

#### Internal Strategy (Model 2)

We found a significant overall impact of *Group* on the use of Internal strategies (*B* = 2.82; *p* = 0.002; adjusted *p* = 0.005). When *Success*, *Change* and *Stress* (i.e., mediators) were added (*CFI* = 1.00, *SRMR* = 0.04) the persons with brain injury still demonstrated significantly greater use of internal aids than the CG (*B* = 1.86; *p* = 0.03; adjusted *p* = 0.005). *Success* was significantly related to the perceived use of internal strategies (*B* = 0.59; *p* < 0.0001; adjusted *p* = 0.003). Conversely, no significant effect was found for *Change* (*B* = 0.06; *p* = 0.67) or *Stress* (*B* = 0.06, *p* = 0.39). No significant indirect effects were found for *Change* (*B* = −0.16; *p* = 0.67) *Perceived Stress* (*B* = 0.09; *p* = 0.43), or *Success* (*B* = −0.71; *p* = 0.08). Neither *Gender* (*B* = −0.54; *p* = 0.60) nor *Age* (*B* = 0.03; *p* = 0.46) were significant.

Altogether, our model indicated that suffering from a brain lesion and having a greater commitment to succeeding in memory tasks were consistent with an increase in the perceived use of internal strategies.

#### Reliance Strategy (Model 3)

We found a significant overall (*B* = 3.63; *p* < 0.001; adjusted *p* = 0.003) and direct effect of *Group* (*B* = 3.13; *p* < 0.001; adjusted *p* = 0.005) on perceived reliance. Overall, persons with brain injury perceived that it relied on others more frequently than the CG. Moreover, although *Gender* was significant (*B* = 1.53; *p* < 0.02; adjusted *p* = 0.005), this was not the case for either *Change* (*B* = 0.16; *p* = 0.17) or motivation assessed on the *Success* scale (*B* = 0.01; *p* = 0.24). No significant effect was found for *Age* (*B* = −0.01; *p* = 0.69) or *Perceived Stress* (*B* = 0.03; *p* = 0.47) (*CFI* = 1.00, *SRMR* = 0.04). Finally, we did not observe a significant indirect effect for *Change* (*B* = 0.42; *p* = 0.22), *Success* (*B* = 0.02; *p* = 0.891), or *Perceived Stress* (*B* = 0.06; *p* = 0.53).

Therefore, the perception of relying on others to overcome memory problems was more prevalent among male participants and brain injured patients.

#### Time Strategy (Model 4)

We observed an overall significant effect of *Group* on the perceived used of the *Time* strategy (*B* = 1.45; *p* = 0.007; adjusted *p* = 0.003). When *Success* and *Change* perceptions (i.e., mediators) were added (*CFI* = 1.00, *SRMR* = 0.04), this effect disappeared (*B* = 0.82; *p* = 0.17). Moreover, *Success* (*B* = 0.12; *p* = 0.05; *p*. adjusted = 0.005) was significantly related with *Time* Strategy. Neither *Stress* (*B* = 0.03, *p* = 0.55), *Change* (*B* = 0.17; *p* = 0.11), *Age* (*B* = −0.007; *p* = 0.79) nor *Gender* (*B* = 0.43; *p* = 0.51) were significant. Finally, no significant indirect effects were observed for *Change* (*B* = −0.44; *p* = 0.16), *Success* (*B* = 0.15; *p* = 0.19), or *Perceived Stress* (*B* = 0.04; *p* = 0.59).

In sum, a high commitment in succeeding triggered people in perceiving themselves as spending much more time than they used to in memorizing. Brain lesion effect disappears when motivation in taken into account.

#### Effort Strategy (Model 5)

An overall significant effect of *Group* was found for *Effort* (*B* = 3.86; *p* < 0.001; adjusted *p* = 0.003). When the mediators *Success* and *Change* were added (*CFI* = 1.00, *SRMR* = 0.04), the presence of brain injury continued to have a significant and positive direct effect on their perceived effort to remember important things (*B* = 3.07; *p* < 0.001; adjusted *p* = 0.005). Moreover, motivation, assessed on the *Success* scale had a significant positive effect (*B* = 0.25; *p* = 0.001; adjusted *p* = 0.005). On the other hand, no significant effect was found for *Perceived Stress* (*B* = 0.07; *p* = 0.17), *Change* (*B* = 0.15; *p* = 0.121), not *Age* (*B* = 0.04; *p* = 0.15), or *Gender* (*B* = 0.69; *p* = 0.35). Finally, no significant indirect effects were observed for *Change* (*B* = 0.37; *p* = 0.26), *Perceived Stress* (*B* = 0.11; *p* = 0.31), or *Success* on *Group* (*B* = 0.31; *p* = 0.11).

Altogether, our results indicated that the presence of a brain lesion as well as higher commitment to succeeding was consistent with the perception of making greater effort than before to memorize information.

## Discussion

Memory is an important function in our daily experience, as it supports our perception of the world and our comprehension of our place in it, that is, our adaptation. It is hard to conceive what life would be without the capacity to remember yesterday, or to make plans for the future. Unfortunately, this is the daily experience of persons with brain injury, who, as reported, must cope with ongoing forgetfulness. Therefore, encouraging resilient behaviors through compensatory strategies is a priority for patients, their families and therapists, and an extensive body of research has examined how to overcome neurocognitive impairments through retraining. However, very few studies have looked at patients’ perceptions of their compensation strategies. Given the lack of understanding of the psychological determinants of such perceptions, we aimed to address the following two questions: (1) Does reported compensation differ between the brain lesion population and similar controls? And, (2) Is the presence of a brain lesion itself sufficient to explain differences in self-reported compensatory patterns?

The originality of our research was to address this issue by testing the adjustment between the data, and our multivariate models of compensatory strategies. We postulated that individual background characteristics were mandatory to explain observed differences between the perceptions of clinical and non-clinical individuals regarding their daily behaviors. The Memory Compensation Questionnaire proved to be a useful and sensitive tool to explore perceptions (Thöne and Walther, 2001; Prigatano and Kime, 2003; Huckans et al., 2010; Cooper et al., 2015).

### Reported Memory Compensation in Clinical and Non-clinical Individuals

The first question that we addressed was whether self-reported compensation differed between persons with brain injury and similar controls. Our clinical experience and previous studies suggested that patterns would differ between the two populations, and this hypothesis was confirmed. Persons with brain injury clearly perceive themselves as developing more internal strategies; relying more on others, taking more time, and making more effort to memorize information than matched control participants (partial η^2^ indicated that effect of brain injury was greatest for *Reliance* and *Effort* scales). However, the two groups did not differ significantly in terms of their use of external aids such as notes or a diary. Arguably, this result is at odds with the observations of Prigatano and Kime (2003), who stated that persons with brain injury reported more reliance on external memory aids than healthy people. In that case, we found that when they were compared with a carefully matched control group (regarding age, education, and gender), their reported patterns only differed marginally from non-clinical individuals.

Regarding general factors, the two groups did not differ significantly in the commitment to maintaining pre-injury performance in memory tasks (the *Success* scale, *p* < 0.07). Finally, brain injury was related to a self-reported increase in aids and strategies used over the past 5–10 years (the *Change* scale). Faced with the challenge of brain injury, individuals perceive a higher awareness of change than control participants in the sense of an increase in the use of compensation strategies to cope with their new situation.

### Differences in Determinants of Memory Compensation Reports for Different Strategies

Going further, we examined whether motivation (fMCQ Success), perception of change (fMCQ Change) and perceived stress (PSS) would partly mediate the effect of *Group* on compensatory strategies (indirect effects). Our results were adjusted for age and gender (covariates). Our initial, overall finding merits specific attention: models differed depending on the tested strategy. Moreover, the commitment in succeeding, that is Success, revealed to be a significant variable that triggered people in perceiving themselves differently for tree strategies: Internal, Time and effort. Finally, perceived stress did not revealed to be a significant variable for any of the five compensation strategies.

#### External Strategies (Model 1)

The use of external strategies consists in arranging the environment to support day-to-day performance. Using a diary, notes or calendars was found to be the most popular strategy, regardless of the group. Age proved to be the best predictor of reported External strategy use. In fact, aging was related to greater use of an environmental support, regardless of the presence of a brain lesion, while the presence of a brain lesion was a poorer predictor than aging. Interestingly, we found that persons with brain injury were no more likely than controls to use a pen and paper to take notes, for example. This finding is in line with available evidence in the domain of aging research (Dixon et al., 2001; Martin et al., 2015). No other direct or indirect effect was found for external compensation, and the presence of a brain lesion only marginally impacted the feeling of the use of an external aid.

Clinical practical guidelines provides recommendations for the use of external aids as compensatory devices for individuals suffering from memory impairments following brain injury. In that context, Sohlberg et al. (2007) recommend to determine the specific parameters to support external aid practice. Age revealed, in our work, to be the mandatory parameters impacting the use of such aids. Put simply, it is of clinical interest to take into account the age of any patient as a predictor of external strategies appropriation. More specifically, youth could be a barrier to the enactment of such strategies. Indeed, younger individuals are less able to set up a diary or take notes. In this context, several clinical implications deserve our attention in order to increase acceptance (Fluharty and Priddy, 1993). First of all, our clinical experience shows that a careful care must be given to the way in which the external aid is introduced (McKerracher et al., 2005): psychoeducation following brain injury for younger patients should present the use of external aids as a widespread practice on general population. Indeed, perceived stigmata and perceived usefulness, as well as a lack of natural daily practice before trauma, might be barriers to acceptance of external aids that draw attention to their problems. Further studies are necessary to explore the specific human factors involved in the reject by younger patients of external aids. Secondly, digital aids have a great potential as a tool to support memory. The use of mobile agendas, to-do lists or note-taking applications seems recommended in order to increase the attractiveness of external compensation methods (Chu et al., 2014). They are acceptable and accessible by younger populations. Nevertheless, we currently have little unambiguous data on their benefit on daily functioning and autonomy (Wong et al., 2017; Christopher et al., 2019).

#### Reliance Strategies (Model 3)

Reliance strategies refer to using others to support performance such as asking someone to remind you to go to a medical appointments. The use of others as a way to compensate was reported less often by both groups, and we found that it was significantly and positively linked to brain lesion and gender. Men tend to solicit the help of others more frequently to palliate their memory deficiencies. In sum, they are more likely to rely on their spouse to help remember to do something than the reverse. Here again, these findings are in line with existing evidence in the field of aging (Dixon et al., 2001; de Frias et al., 2003). Special attention should therefore be given to men living alone as they will be unable to implement this natural strategy. Women perceived themselves as underusing the assistance of others to compensate for their difficulties. Results from the aging literature are of interest. Some personality dispositions, specifically the locus of control for women, revealed to contribute to general compensation in late life (de Frias et al., 2003). In women, a high locus of control is protective of becoming depressed when experiencing cognitive impairments (van den Heuvel et al., 1996). More generally, Ziff et al. (1995) found that perceived control in young and middle-aged adults is associated with the degree to which one’s is involved in health promoting behaviors. Individuals with a high locus of control might be associated with the belief they are in control with the situation and that their success is linked to their own efforts. To anticipate on our results, women with a high locus of control might not be motivated to use strategies such as external aids if they perceive them out of their control. Further investigations are needed to explore the link between gender, locus of control and cognitive compensation. In any case, it is actually not known whether engaging in external strategies would help women improve their daily functioning. Nevertheless, it seems useful to get women to seek help from others, especially when implementing digital compensation tools. Indeed, studies show that the effectiveness of technological aids depends on the accessibility to human technical support (i.e., Wong et al., 2017). Women could thus abandon their use for lack of spontaneous recourse to others. But, here again, we currently have little data dealing with the link between external memory compensation and digital tools usage.

Altogether, our results highlight that socio-demographic variables (age and gender) play an important role in the self-report use of strategies that rely little on deliberative cognitive processes, that is external and reliance strategies.

#### Internal Strategies (Model 2), Time (Model 4), and Effort (Model 5) Strategies

Our data reveals close patterns for Internal, Time and Effort strategies. Internal strategies rely heavily upon self-control and deliberative processes, and do not draw upon environmental support. *Effort* and *Time* strategies refer to paying attention, engaging effort, doing one’s work well, and tenacity. We found that the commitment to succeed in memory tasks positively impacted the self-reports of those tree types of compensation strategies. In other words, motivation (*Success*) was found to be a cognitive bias that mediated the impact of the lesion; and even, concerning the *Time Strategy*, brain lesion effect disappeared when motivation was introduced in the model.

Further investigations are needed to understand and explain this common pattern. To anticipate, putting in effort and taking extra time to complete a task are socially highly valued responses. Indeed, from school age, teachers and parents encourage children to try and work hard. Turner (1998) found that 95% of respondents thought that effort was almost the only cause of academic and life outcomes, in other words the major determinant of success or failure. Consequently, extra effort results in a feeling of pride and accomplishment (Skinner et al., 1990; Lewis, 2000). There is a general belief that effortful strategies are the best way to improve cognitive functioning. Further works are needed to explore whether brain-injured individuals endorse this societal belief, leading them to self-report more effortful strategies. If this is the case, self-reports may prove to be based upon socially introjected beliefs and goals, rather than reflecting true self-regulation (e.g., Ryan and Stiller, 1991; Ryan et al., 1997). In any case, in the rehabilitation context, a high level of motivation could prove to be an obstacle to the implementation of new external compensatory behaviors: individuals who consider effortful strategies to be most valuable might be resistant to behavioral change (Borkowski et al., 1986). In the context of the emotional experience of loss, it is essential to consider victims’ awareness and beliefs about the consequences of their injury (Kit et al., 2007; Garrett et al., 2010). Indeed, their assessment of the relevance and effectiveness of a compensation strategy might not be based on the evaluation of past, objective performance but on desirable, future behaviors. Another interesting direction already mentioned above is the impact of the locus of control (LOC) on self-referent beliefs. Rotter (1966) characterized the LOC as a personality trait that is stable over time even if it can vary with circumstances. According to his theory, the achievement of a result is conditioned by the link that individuals perceive between their own actions and this result. Some individuals would perceive outcomes as determined by their own actions or behaviors (i.e., internal locus of control) while others would perceive them as determined by external factors (i.e., external locus of control). Zahodne et al. (2018) demonstrated that non-demented older adults, with stronger control beliefs, maintained memory function in the face of lower hippocampal volume. Moreover, the authors showed that this impact of control beliefs is stronger for cultural minorities. They concluded that culturally appropriate interventions are needed to test whether the locus of control provides or not a resistance to cognitive decline. Moore and Stambrook (1992) found a link between the LOC and mood disturbance following TBI (see also Finset and Andersson, 2000). Therefore, the link between locus of control, emotional and memory capacity is now relatively well established. New studies are needed to explore whether this link also exists with self-referent beliefs about memory compensation, especially in the field of cerebral damage.

The current study aimed to contribute to the field of memory compensation strategies literature by investigating some determinants of self-reference beliefs after TBI in order to minimize resistance to treatment. Additional research is recommended due to several limitations. First of all, we opted for broad inclusion criteria of our population which led to a heterogeneity of diagnosis which complicate interpretations. For that reason, we estimated the effect of the types of brain injuries on our results. We found that the random effects for diagnosis could be removed from our subsequent analyses. Second, the use of the Glasgow scale to characterize the severity of the injury as “mild” is controversial due to its lack of prognostic utility and its poor inter-rater reliability. At the time of patients’ inclusion, the physician referred to the DSM4 criteria to confirm the diagnosis of mild brain injury [American Psychiatric Association (APA), 1994, 2000]. A valid criticism that can be made regarding these criteria is that the diagnosis rested on the initial severity of TBI regardless of effects on everyday functioning (Wortzel and Arciniegas, 2014). In order to ensure the diagnosis of mild injury, we included only patients for whom cognitive deficits did not interfere with their ability to be independent in the activities of daily living. Third, only one measure was used for each construct of interest. Thus, all the interpretations and conclusions are based upon the validity of each scale. It would be interesting to see if the results remain consistent using additional measures. Finally, further investigations may examine the correlation between the ratings on the compensation questionnaire and the patients’ everyday performance as we did not have external and independent indicators of everyday memory behaviors. For all these limitations, our results should be considered as preliminary.

To conclude, human resilience when faced with memory deficiency is a complex process that requires consideration of multiple levels of analysis to understand reported compensation in different settings. Compensatory approach are ways of bypassing for impaired functions and behavioral changes that are multiform. The design of compensation training needs to consider variability in patients’ self-referenced beliefs to be effective. Beliefs vary among both persons and types of compensation strategy, and have the potential to either empower or inhibit day-to-day behavior. As a result, therapists should take care to consider the different determinants at work when choosing a rehabilitation program, as different strategies differ in terms of their emotional and sociodemographic determinants. In turn, these determinants impact patients’ perceptions of compensation styles, which subsequently influence their behavioral choices. This is especially true when therapists work with brain injury victims who suffer from catastrophic thinking, and fail to distinguish between an ordinary error and an error caused by the injury (Mathias and Coats, 1999; Kit et al., 2007). A personalized approach would allow patients to be more compliant and to prevent the daily non-use of strategies set up with the therapist. For example, it is valuable to work on the attractiveness of external strategies to promote its use; instead of putting the light on its efficacy to decrease troubles and distress. It is interesting to question the patient on the “why” of the use of such or such strategy, rather than being interested only in the quantification of its daily use.

In a study that is similar to ours, Huang et al. (2014) noted that brain-injured persons might also be victims of a social expectancy effect and endorse others’ metacognitions about the severity of their memory failure. It is possible that victims accept the injunctions of society and therapists in coping with their memory failures across the entire spectrum of daily activities. Such injunctions may influence how patients perceive and, consequently, assess day-to-day strategies. In particular, they may overestimate post-concussion compensatory changes in a manner that is consistent with expectancies in their environment. In this context, as Prigatano (1999) and Wilson (2008) state, memory rehabilitation will fail if neuropsychologists do not deal, first and foremost, with the emotional and metacognitive consequences of TBI, rather than cognitive efficiency. We thus agree with the statement of Trexler (2000), according to whom new paradigms for rehabilitation of persons with brain damage must embrace experiential aspects of rehabilitation. A more accurate picture of how brain-injured persons perceive compensation strategies would benefit from research that supplements performance and self-reporting with naturalistic observations of people’s use of compensation strategies in their everyday setting.

## Data Availability Statement

The raw data supporting the conclusions of this article will be made available by the authors, without undue reservation.

## Ethics Statement

Ethical approval was not provided for this study on human participants because ethical approval was not necessary as (1) our study was not invasive, (2) all participants gave their consent, (3) none of the participants can be identified, and (4) our laboratory committee validated the study. The patients/participants provided their written informed consent to participate in this study.

## Author Contributions

SM was responsible for the research axis and the principal writer of the article. RT analyzed data with the R package lavaan and wrote the corresponding results section. RT, DB, LT, and EG-N contributed to the theoretical aspects. SM and CM participated in data collection. DM assisted in the correction of the English language. All authors contributed to the article and approved the submitted version.

## Conflict of Interest

The authors declare that the research was conducted in the absence of any commercial or financial relationships that could be construed as a potential conflict of interest.
